# The FOUND questionnaire: identifying stable traits associated with success in remote operations—an exploratory study

**DOI:** 10.3389/fnsys.2025.1676412

**Published:** 2025-11-06

**Authors:** Valentina Cesari, Enrico Cipriani, Giorgia Papini, Andrea Piarulli, Angelo Gemignani, Danilo Menicucci

**Affiliations:** Department of Surgical, Medical and Molecular Pathology and Critical Care Medicine, University of Pisa, Pisa, Italy

**Keywords:** personality, traits, telemanipulation, embodiment, skills learning, dexterity

## Abstract

**Introduction:**

This study introduces the FOUndatioNal trait-BaseD Characterization (FOUND) questionnaire, specifically developed to assess stable characteristics of perceptual, cognitive, and emotional domains associated with effective performance in mediated settings.

**Materials and methods:**

Items were derived from interviews with professionals in remote technology fields (e.g., robotic surgeons, drone pilots, crane operators) and grouped into four domains: cognitive-behavioral, socio-emotional, functional-organic, and value-based. Items, rated on a 4-point Likert scale, were designed to reflect stable traits. A panel of nine experts evaluated content validity; items with a CVI > 0.78 and a mean relevance ≥3 were retained, resulting in a 26-item scale. The factorial structure of FOUND was validated in a sample of 300 Italian participants, with convergent validity assessed, and participants were subsequently categorized into high and low procedural skill professions for known-groups comparisons (Study 1). Additionally, a separate sample of 34 remote operators (Study 2) was included to further evaluate known-groups validity.

**Results:**

Exploratory and confirmatory factor analyses yielded a final 22-item structure, identifying four factors: Perception and Action, Empathic Attitude, Stress Management, and Group-Oriented Values. Convergent validity analysis using questionnaires that assess personality and stable characteristics (Five Facet Mindfulness Questionnaire-15, Big Five Inventory-10) did not yield significant correlations, indicating that the FOUND questionnaire may provide independent information. Known-groups validity was assessed by comparing scores between professions requiring high and low procedural skills identified in the 300 participants, revealing higher scores in Perception and Action, Empathic Attitude, and Stress Management for the first group (Study 1). Comparing remote operators (i.e., drone pilots) with the general population showed that remote operators scored higher in Perception and Action and Group-Oriented Values but lower in Stress Management, highlighting distinctive characteristics of individuals engaged in remote operations (Study 2).

**Conclusion:**

The FOUND assesses perceptual, motor, cognitive, and socio-emotional constructs associated with performance in mediated and remote operations. It allows evaluation of stable traits and performance-related attitudes in contexts such as robotic surgery, telemedicine, education, and emergency response. By identifying these traits, the questionnaire can inform the design of personalized interventions and training programs tailored to individual characteristics, enhancing effectiveness in mediated environments.

## 1 Introduction

The growing reliance on advanced telecommunication systems has driven the adoption of immersive technologies and remote control interfaces across several professional domains. These systems enable the remote performance of actions, such as robotic surgery or drone piloting, and highlight the importance of identifying individual characteristics associated with effective operation in such mediated environments.

The use of immersive scenarios (e.g., virtual reality) and remote control manipulation involving simple joysticks or complex robotic effectors in professional settings such as robotic surgery and drone control has been shown to influence users’ perceptual, emotional, and cognitive processes, with studies predominantly focused on human-interface interaction in context-specific ways (Cesari et al., 2024). As an illustrative example, motor learning in virtual environments is slower and less accurate than in real-world contexts (Magdalon et al., 2011). Remote manipulation, such as robotic-assisted surgery, often lacks important sensory inputs like tactile feedback, which can hinder performance. These disruptions may stem from limited experience, spatial uncertainty, reduced visual fields, or visuomotor distortions (Magdalon et al., 2011; Menicucci et al., 2020). Together, these factors can disrupt the sense of embodiment—a crucial element in integrating external tools into one’s body schema in such environments (Kilteni et al., 2012; Toet et al., 2020).

Given the growing prevalence of mediated and remote environments, there is an increasing need for models that capture stable, domain-specific individual influencing performance across contexts. This supports a trait-based perspective in human–computer interaction research. Traits are enduring patterns of behavior, thought, and emotion. They influence how individuals perceive and act in different environments and may help identify those who adapt more effectively to remote operational demands (McCrae and Costa, 1999; Jayawickreme and Zachry, 2020), beyond situational training or skill-based models.

In spite of this, it is crucial to differentiate traits from skills or states. Traits are relatively stable predispositions that shape how a person typically thinks, feels, and behaves, while skills and states reflect what a person is capable of doing or experiencing in specific situations. Although related, what individuals tend to do (traits) and what they can do (skills) are not always perfectly aligned—a distinction often overlooked in current research (Ringwald et al., 2025).

It has been emphasized that a clear conceptual distinction between traits and states is essential for understanding human performance (Brunyé et al., 2024). Traits are relatively enduring characteristics that differentiate individuals and influence outcomes, while states are temporary and context-dependent; however, authors stated that traits alone may not fully account for performance variability (Brunyé et al., 2024). Interestingly, prolonged or repeated motivational states—such as diligence, organization, or goal-directed effort—when experienced repeatedly in daily life, can gradually consolidate into stable personality traits (Costantini et al., 2020). This highlights the importance of integrating both enduring traits and dynamic states when modeling individual differences in performance.

Building on this distinction, professions also differ in their procedural demands, that is, in the complexity and precision of action sequences required for task completion. In this context, procedural knowledge refers to “knowing how” to perform actions or tasks, usually acquired through experience and practice, and is often difficult to verbalize (Newell and Simon, 1972; Anderson, 1976). It encompasses motor, cognitive, and habit-based skills, stored as condition–action or stimulus–response associations, which guide behavior without requiring conscious awareness (Knowlton et al., 2017).

High procedural skill professions, such as doctors, engineers, architects, and managers, require precise execution of structured sequences of actions and rely on both tacit and procedural knowledge acquired through practice and experience (Martin, 1987; Cegarra-Navarro et al., 2017; Green et al., 2022; Dissaux and Jancart, 2023). On the other hand, low procedural skill professions, such as office employees, sales personnel, or agricultural workers, involve less cognitively and motorically complex tasks, relying mainly on explicit knowledge (Smith, 2001; Voronchuk and Starineca, 2014; Groza and Groza, 2018).

This distinction is also critical in mediated and remote operational environments, where high procedural skill professions not only require technical competence but also cognitive, emotional, and perceptual skills appear to be influential. For example, in robotic surgery, successful performance depends on integrating sensory information, managing stress, collaborating in teams, and quickly adapting to dynamic scenarios (Hagen et al., 2008; Enayati et al., 2016).

According to the extant scientific literature, a small body of studies has attempted to investigate the personality traits that shape the interplay between humans and interfaces, relying primarily on three distinct domains of inquiry: (1) illusion-based paradigms (e.g., body malleability), (2) action-oriented telemanipulation tasks, and (3) perception of personality in virtual or robotic agents. The first domain is represented by research on body malleability, a key feature in telemanipulation due to the prominent role of extending body boundaries to incorporate robotic and virtual extensions. In these cases, the Rubber Hand Illusion has been widely used as a gold standard paradigm (Botvinick and Cohen, 1998). For instance, Burin et al. employed the Personality Assessment Inventory and the Rorschach test, thereby finding a significant association between the perception/self-representation domains and illusory hand mislocalization (Burin et al., 2019). From a cognitive perspective, Yeh et al. found that participants with lower switch costs and higher attention-shift scores had faster illusion onset times and that those with higher attention-shift scores experienced the illusion more vividly (Yeh et al., 2017). Finally, another crucial study, performed by Perepelkina showed that higher emotional intelligence might improve multisensory integration of body-related signals and reflect better predictive models of self-processing (Perepelkina et al., 2017).

The second domain concerns action-oriented telemanipulation tasks. However, unlike research on personality in illusion paradigms, these works used brief questionnaires to assess stable traits, primarily those of the Big Five model: openness, conscientiousness, extraversion, agreeableness, and neuroticism (McCrae and Costa, 1987; Goldberg, 1990, 1993). In this regard, several noteworthy findings show that personality traits play a significant role in influencing subjective perception, performance, and technology adoption, thus contributing to understanding the interplay between humans and computer technology. In this vein, Muller et al. found that individuals with lower neuroticism were more likely to appreciate robotic co-manipulation arms in terms of comfort and usefulness (Muller et al., 2022). Qin and colleagues demonstrated that extroversion increased collision rates under latency, while neuroticism led to performance delays due to anxiety (Qin et al., 2022).

The third domain pertains to the perception of personality in virtual or robotic agents. Wang and colleagues showed that hand motion attributes significantly shape the perception of a virtual character’s personality, with different movements influencing extraversion, openness, and neuroticism (Wang et al., 2016).

Collectively, these studies demonstrate that personality traits can impact performance in various professional and learning environments. It should be noted, however, that most of these questionnaires or tasks did not take into account the specificity of telemanipulation. For example, none of the aforementioned measures considers the putative intersubjective and stable differences in body malleability when using telemanipulation, thus underestimating the potential role of the sense of embodiment. Importantly, teleoperations require strong group coordination, as seen in robotic surgery, where seamless communication between remote operators and surgical team members is crucial. Traditional personality models, like the Big Five, assess traits like Agreeableness and Conscientiousness but do not capture structured teamwork skills, hierarchical adherence, or the ethical-motivational aspects of collaboration. Similarly, empathy measures focus on understanding emotions rather than teamwork dynamics. Stress scales like the Perceived Stress Scale (PSS) (Cohen et al., 1983) measure individual stress but not stress regulation in team settings. A more integrated approach is needed to assess both individual cognitive skills and their interaction within group dynamics for optimal coordination in high-demand remote tasks.

It is worth noting the overlooked involvement of cognitive studies in shaping the interplay between humans and interfaces, with several contributions emphasizing the importance of individual cognitive skills as key determinants of task performance in telemanipulation contexts. For example, Guru et al. showed that expert surgeons allocate mental resources differently depending on task complexity, with both cognitive performance and motor execution contributing to workload (Guru et al., 2015). Johnsen assessed cognitive skills in police drone pilots, finding that spatial orientation and attentional selection were the strongest predictors of task proficiency (Johnsen et al., 2024). Cesari et al. explored cognitive engagement in online learning, underscoring the role of flow and presence in virtual environments (Cesari et al., 2021). While these studies highlight important situational skills and cognitive states, it is equally important to investigate the underlying cognitive traits that drive consistent patterns of behavior across contexts. Individual differences are, in fact, multifaceted: while experience and bias drive our learned behaviors and cognitive states capture our temporary mental conditions, it is cognitive traits—enduring tendencies to respond consistently to certain types of stimuli or situations—that represent the most foundational aspect (Ottley, 2020).

Building on these considerations, it is crucial to identify stable traits that influence how humans interact with interfaces, independently of their specific skills. To this end, we developed a questionnaire to operationalize a trait-based framework, aiming to detect enduring psychological characteristics associated with effective performance in technologically mediated environments, such as robotic surgery, drone operation, or remote collaboration. Addressing the limitations of existing measures, we introduce a newly designed instrument that integrates key constructs from the perceptual, motor, cognitive, and emotional domains into a single tool. We have named this self-report questionnaire FOUndatioNal trait-BaseD Characterization (FOUND).

To more effectively capture individual differences in stable traits, FOUND has been conceived as a composite questionnaire grounded in a multidimensional structure—encompassing cognitive-behavioral, socio-emotional, functional-organic, and value-based dimensions—associated with the complexity of individual variation relevant to human–interface interaction. To enhance its theoretical coherence, this structure aligns with established trait taxonomies such as the Big Five (McCrae and Costa, 1987), the HEXACO model (Ashton and Lee, 2007), and Cattell’s 16 Personality Factors (16PF) (Cattell, 1946). To illustrate, socio-emotional and value-based domains show a convergence with traits like Agreeableness, Honesty-Humility, and Emotionality (HEXACO), or Warmth, Sensitivity, and Rule-Consciousness (16PF). Similarly, cognitive-behavioral traits closely align with Conscientiousness and Openness to Experience (Big Five), as well as with Reasoning and Abstractedness (16PF). The functional-organic domain—encompassing stress management, fatigue, and circadian rhythms—closely resonates with Neuroticism (Big Five), Emotional Stability (16PF), and broader constructs of physiological resilience and self-regulation that appear to be crucial in occupational settings. Thus, positioning the FOUND questionnaire within these theoretical frameworks strengthens its conceptual foundation and highlights its relevance across both psychological research and applied performance contexts. Grounding FOUND in trait theory is important to prevent the limitation of considering traits in isolation and the potential influence of dynamic states on performance (Brunyé et al., 2024). Importantly, repeated or prolonged experiences reflected in certain states may become integrated into enduring dispositions (Costantini et al., 2020), reinforcing the importance of assessing both stable traits and their interactions with situational factors to understand human performance in complex mediated environments.

Item selection and domain identification were guided by integrative theoretical frameworks, enabling the characterization of stable individual differences and task-relevant competencies across four key domains of individual functioning (i.e., cognitive-behavioral, functional-organic, socio-emotional, and values-based).

Building on this theoretical framework, we conducted two studies: the first focused on validating FOUND and investigating known-groups validity between high and low procedural skill professions, while the second examined known-groups validity comparing the general population with individuals engaged in remote manipulation tasks. In Study 1 we hypothesized that: (1) FOUND would display a four-factor latent structure corresponding to the aforementioned domains (i.e., cognitive-behavioral, functional-organic, socio-emotional, and values-based); (2) it would show moderate convergent validity with established personality domains, including Agreeableness, Extraversion, Emotional Stability, Conscientiousness, as measured by the Big Five Model (Roccas et al., 2002) and Acting with Awareness and Observing from the Five Facet Five Facets Mindfulness Questionnaire (Baer et al., 2012); and (3) individuals engaged in professions requiring higher procedural abilities would score higher across all four FOUND domains compared to those in lower procedural skill professions.

In Study 2, we extended this framework to remote manipulation operators, hypothesizing that these individuals would demonstrate enhanced functioning in cognitive-behavioral domains relative to the general population. We placed particular emphasis on perceptual abilities, including body malleability, spatial orientation, and the capacity to infer tactile or haptic information from visual cues, which are critical for precise remote control and rapid multisensory integration in technology-mediated environments.

## 2 Study 1

In Study 1, the FOUND was administered to a sample of Italian participants. This study was designed to evaluate the scale’s factor structure, its convergent validity with established personality and mindfulness measures, and its ability to detect differences related to professional skill demands (known-group validity).

### 2.1 Scale development and initial validation

We designed our questionnaire to include items belonging to the following domains:

1. Cognitive-behavioral (assessing cognitive processes and behaviors such as problem-solving, decision-making, and situation awareness).
2. Socio-emotional (interaction with other people, prosocial behaviors, empathic attitude).
3. Functional-organic (stress management, physical strain, fatigue, circadian rhythms).
4. Value-based (social responsibility, ethical, and moral considerations).

Draft items were developed based on insights from 10 informal, unstructured interviews conducted with individuals working with mediated technologies, including robotic surgeons, crane operators, and drone pilots. The interviews were focused on their experiences, challenges, and perceptions.

The emerging themes from the interviews were manually transcribed and categorized according to four overarching domains we had conceptually defined (i.e., cognitive-behavioral, socio-emotional, functional-organic, and value-based). Draft items were then generated by rephrasing recurring phrases and concerns into declarative statements reflecting dispositional tendencies (e.g., “I perceive my body as flexible and adaptable” or “I consider obedience to be an important personal value”). Each item, conceived to avoid time-bounded phrasing to emphasize stable traits rather than situational states, required an answer based on a 4-point Likert scale to express the individual’s agreement (1 = strongly disagree; 2 = disagree; 3 = agree; 4 = strongly agree).

To obtain the final scale, the draft items were submitted to a panel of experts for a content validity evaluation, in line with Lynn (1986). We contacted 9 experts in remote manipulation activities and jobs (drone piloting, robotic surgery). These experts were asked to rate each draft item for relevance in measuring the extent to which an individual exhibits stable traits across the four key domains, and their importance in remote operations: cognitive behavioral, socio-emotional, functional organic, and values-based domains. Relevance was measured on a 4-point scale ranging from “Not relevant” to “Very relevant.” The 4-point score was averaged between the experts, thus obtaining the Content Validity Index (CVI), a mean measure of relevance for each item. In addition, the CVI was re-coded into a dichotomous “Relevant/not Relevant” scale (scores 1 and 2 as “Not relevant,” and 3 and 4 as “Relevant”), and computing the percentage of experts who rated each item as “Relevant,” thus obtaining a measure of expert agreement. For the following phases, we retained only those items exceeding a CVI cutoff of 0.78 (Lynn, 1986) and having a mean score of 3 or higher. This procedure resulted in a 26-item scale submitted to the next validation phases.

#### 2.1.1 Participants

A total of 315 individuals from the Italian population were initially enrolled for Study 1, in line with best practices for psychometric instrument evaluation (Boateng et al., 2018; Tabachnick and Fidell, 2019). Participants were recruited via the Prolific.com online platform (Palan and Schitter, 2018) and received monetary compensation of £1.5 each. The median time to complete the survey was 15 min.

To ensure data quality, the survey included five attention check items instructing participants to select a specific response (e.g., “Please select ‘4’ for this question”). Participants who failed more than one attention check, as well as those who did not provide informed consent, were excluded from the analyses.

After these exclusions, the final analyzed sample comprised 300 participants (150 males, 150 females; age: *M* = 32.4 years, SD = 10.2, range = 18–65; attrition rate: 4.76%).

For factor analyses, this sample was randomly split into two non-overlapping subsets: 200 participants (100 males, 100 females) for the Exploratory Factor Analysis (EFA) and 100 participants (50 males, 50 females) for the Confirmatory Factor Analysis (CFA).

All participants received a detailed protocol briefing before completing the questionnaire and were debriefed regarding the study objectives after completion. Age was later controlled for in regression analyses to account for potential confounding effects.

The survey study protocol and its contents were approved by the Bioethical Committee of the University of Pisa on 28/01/2022 (n.3/2022).

#### 2.1.2 Materials

The survey included four sections:

1. Collection of demographic information, such as age, gender identification, level of education, and occupation, to characterize the sample and allow for potential group comparisons.
2. Administration of the Five Facet Mindfulness Questionnaire-15 (FFMQ-15) to assess convergent validity by examining the extent to which FOUND dimensions align with or diverge from established mindfulness traits. The FFMQ-15 was selected due to mindfulness being a pivotal trait underpinning attentional control, cognitive flexibility, and emotion regulation, key competencies associated with effective functioning in technology-mediated, high-demand environments.
3. Administration of the Big Five Inventory-10 scale (BFI-10) to assess the convergent validity by evaluating whether FOUND dimensions overlapped with or diverged from established personality traits, to gain knowledge about complementary insight into the relationships between FOUND and broader personality traits.
4. Administration of the FOUND questionnaire for validation.

##### 2.1.2.1 Big Five Inventory-10 (BFI-10)

The BFI-10 is a 10-item scale measuring five personality traits: Extraversion, Agreeableness, Conscientiousness, Neuroticism, and Openness (Rammstedt and John, 2007). Test–retest correlations suggest an acceptable reliability of the scale. All items are rated using a 5-point Likert scale ranging from 1 (strongly disagree) to 5 (strongly agree). Factor analysis revealed a five-factor solution consistent with the Big Five factors (Openness to experience, Agreeableness, Extroversion, Neuroticism, and Conscientiousness), with each item loading on the intended factor. Correlations with other Big Five instruments, correlations between self and peer ratings, and associations with sociodemographic variables suggest a good validity of BFI-10 scores.

###### 2.1.2.2 Five facets mindfulness questionnaire-15 (FFMQ-15)

The Five Facet Mindfulness Questionnaire (FFMQ-15) is a 15-question self-report scale that assesses mindfulness regarding thoughts, experiences, and actions in daily life (Baer et al., 2012). The FFMQ-15 measures 5 subscales of mindfulness: (1) Observing, the way the individuals can see, feel, and perceive the internal and external world around us and select the stimuli that require our attention and focus; (2) Describing, the way we label our experiences and express them in words to ourselves and others; (3) Acting with Awareness: the actions we choose after attending to the information present at the moment; (4) Non-judgment, the ability to be non-judgmental regarding our inner experience; (5) Non-reactivity, active detachment from negative thoughts and emotions to accept their existence and choose not to react to them.

The results of the FFMQ-15 are the total average score and the scores of the five subscales. Higher scores are indicative of someone who is more mindful in his/her everyday life.

The scores of the five subscales of the FFMQ-15 give a reliable measure of mindful self-awareness. The FFMQ-15 is useful to determine whether individuals who practice mindfulness become more mindful over time or whether low mindfulness could affect psychological health.

#### 2.1.3 Data analysis

The factor structure of the FOUND was examined using parallel analysis, EFA, and CFA on separate samples, with reliability assessed via Cronbach’s *α*. Convergent validity was evaluated through correlations with the FFMQ-15 and BFI-10. Known-groups validity was tested by comparing FOUND scores across occupations differing in procedural demands, using repeated-measures ANOVAs, post-hoc tests, and effect size estimates.

All data manipulation and statistical analyses were conducted in R (ver. 4.2.0) within RStudio. EFA, parallel analysis, CFA, and reliability estimates were computed using the “psych” package (ver. 2.3.6; Revelle, 2015) and (ver. 0.6–15; Rosseel, 2012).

##### 2.1.3.1 Factor analysis

The factor structure was explored through a parallel analysis (Hayton et al., 2004) and EFA. For the EFA, we used the “MinRes” estimator with “oblimin” factor rotation on 200 participants (100 males and 100 females), selected using a random seed. Scale items were excluded if they exhibited significant cross-loadings (loadings ≥ 0.32 on two or more factors). The EFA process was iterated on the reduced scale until a satisfactory factor structure was attained.

Reliability estimates (Cronbach’s *α*) were computed for each factor. A CFA using the Weighted Least Squares Mean and Variance adjusted (WLSMV) estimator and Satorra and Bentler (1994) correction was conducted (Li, 2016) on the disjointed set of the remaining 100 subjects (50 males and 50 females). The CFA was conducted to test the structures of latent factors obtained via EFA, thus ensuring that the model accurately reflects the relationships between items and the underlying constructs (latent factors).

##### 2.1.3.2 Convergent validity

Convergent validity was assessed by examining the correlation matrix among the scores of FOUND, FFMQ-15, and BFI-10. To evaluate the convergent validity of FOUND, correlations were calculated with both FFMQ-15 and BFI-10. Correlations with FFMQ-15 provided primary evidence of convergent validity, while correlations with BFI-10 offered complementary insights into the relationships between FOUND and broader personality traits.

##### 2.1.3.3 Known-groups validity

The evaluation of known-groups validity was conducted through a comparative analysis of FOUND scores among participants in occupations characterized by high and low procedural ability. Within each broad category, participants were further divided into subgroups: High Procedural included Creative Service, Professional Management, and Technical professions, while Low Procedural included Administrative and Field-based professions. Type III Repeated-Measures ANOVAs were conducted both within each broad category to assess potential differences among subgroups, and across the aggregated High Procedural versus Low Procedural groups. FOUND factors (Perception and Action, Empathic Attitude, Stress Management, and Group-Oriented Values) were treated as the within-subjects factor. Partial eta-squared (*η*^2^*ₚ*) values were calculated to estimate the proportion of variance explained by the main effects and interaction. Additionally, post-hoc Bonferroni-corrected pairwise comparisons were conducted both between High Procedural and Low Procedural groups and within their respective subgroups for each FOUND factor. For these comparisons, Cohen’s *d* was calculated to quantify the magnitude of the differences. To compute known-groups validity, we excluded students and retired individuals.

### 2.2 Results

#### 2.2.1 Factor structure of the FOUND questionnaire: a four-factor model

To assess the adequacy of our final survey sample for factor analysis, we conducted both the Kaiser-Meyer-Olkin (KMO) measure and Bartlett’s test of sphericity. The results showed a KMO value of 0.70 and Bartlett’s test statistic of 1008.73 with a *p*-value ≤ 0.001. Additionally, parallel analysis with 1,000 iterations and examination of the scree plot indicated that four latent factors should be retained. Based on the item loadings, we decided to retain 22 items and to label the factors “Perception and Action” (e.g., “I consider myself more skilled than others at orienting myself in space.”), “Empathic Attitude” (e.g., “I believe that every person deserves care and love.”), “Stress Management” (e.g., “Even when my workload is high, I manage not to get disturbed during my leisure time.”), and “Group-Oriented Values” (e.g., “In a group, it is necessary to have a leader who directs everyone’s work.”) (Table 1).

**Table 1:** Standardized factor loadings from the Exploratory Factor Analysis (EFA).

Item	f1	f2	f3	f4	Unique Var	Communalities
Found 1	.	.	.	0.44*	0.81	0.19
Found 2	0.50*	.	.	.	0.71	0.29
Found 3	0.33*	.	.	.	0.72	0.28
Found 4	.	0.45*	.	.	0.77	0.23
Found 5	.	0.48*	.	.	0.76	0.24
Found 6	0.56*	.	.	.	0.63	0.37
Found 7	.	0.59*	.	.	0.60	0.40
Found 8	.	.	.	.	0.91	0.09
Found 9	.	.	−0.54*	.	0.65	0.35
Found 10	0.35*	.	.	.	0.68	0.32
Found 11	.	.	.	.	0.96	0.04
Found 12	.	.	0.39*	.	0.83	0.17
Found 13	.	.	.	.	0.91	0.09
Found 14	.	.	.	0.36*	0.83	0.17
Found 15	.	0.81*	.	.	0.36	0.64
Found 16	0.33	.	0.37*	.	0.62	0.38
Found 17	0.52*	.	.	.	0.70	0.30
Found 18	.	0.58*	.	.	0.64	0.36
Found 19	.	.	.	0.34*	0.82	0.18
Found 20	.	0.35*	.	.	0.74	0.26
Found 21	.	.	.	0.56*	0.68	0.32
Found 22	.	.	0.43*	.	0.62	0.38
Found 23	.	.	.	.	0.92	0.02
Found 24	.	0.67*	.	.	0.43	0.57
Found 25	.	.	0.48*	.	0.76	0.24
Found 26	.	.	0.35*	.	0.74	0.26

Loadings marked with * are significant at the 0% level. A dot (.) indicates no substantial loading on that factor. Unique variances and communalities for each item are also reported.

Results of CFA showed a good fit for all 4 factors extracted (Robust CFI = 0.924; Robust TLI = 0.913; Robust RMSEA = 0.52; 90% CI [0.033, 0.060]; SRMR = 0.057) (Table 2).

**Table 2:** Standardized factor loadings from the Confirmatory Factor Analysis (CFA).

Item	Factor	Loading	SE	*z*-value	*p*
Found 2	Perception and action	1.000	–	–	–
Found 3	Perception and action	1.379	0.259	5.320	0.000
Found 6	Perception and action	0.902	0.183	4.939	0.000
Found 10	Perception and action	0.999	0.174	5.728	0.000
Found 17	Perception and action	0.832	0.177	4.707	0.000
Found 4	Empathic attitude	1.000	–	–	–
Found 5	Empathic attitude	1.514	0.342	4.430	0.000
Found 7	Empathic attitude	2.215	0.371	5.965	0.000
Found 15	Empathic attitude	2.433	0.409	5.949	0.000
Found 20	Empathic attitude	2.698	0.472	5.717	0.000
Found 24	Empathic attitude	2.554	0.449	5.692	0.000
Found 9	Stress management	1.000	–	–	–
Found 16	Stress management	−2.207	0.608	−3.626	0.000
Found 18	Stress management	−1.808	0.429	−4.216	0.000
Found 22	Stress management	−2.085	0.572	−3.646	0.000
Found 25	Stress management	−1.421	0.422	−3.370	0.001
Found 26	Stress management	−1.616	0.437	−3.699	0.000
Found 1	Group-oriented values	1.000	–	–	–
Found 12	Group-oriented values	1.083	0.341	3.172	0.002
Found 14	Group-oriented values	1.269	0.380	3.336	0.001
Found 19	Group-oriented values	0.827	0.364	2.269	0.023
Found 21	Group-oriented values	2.011	0.545	3.690	0.000

Each latent factor’s first item loading is fixed to 1.0 to identify the model; therefore, no standard error, z-value, or p-value is reported for these reference items. All other loadings are estimated relative to this reference item. Negative loadings indicate that the item is inversely related to the latent factor.

Descriptive statistics for the four FOUND factors are reported in Table 3. The final Italian and English versions of the FOUND questionnaire, together with its factorial structure, are presented in Supplementary material 1, which includes: 1.1. FOUND questionnaire – Italian version, 1.2. FOUND questionnaire – English version, and 1.3. FOUND questionnaire – Items and Factor Structure.

**Table 3:** Descriptive statistics of the FOUND Factors derived from the sample of the Italian population.

Factor	Mean	Standard deviation
Perception and action	2.45	0.54
Empathic attitude	3.27	0.46
Stress management	2.66	0.53
Group-oriented values	2.69	0.44

#### 2.2.2 Reliability: FOUND factors show fair-to-acceptable internal consistency

Internal consistency estimates for the FOUND subscales, as derived from the CFA solution, ranged from low to acceptable. Specifically, Perception and Action showed a Cronbach’s *α* of 0.69, Empathic Attitude *α* of 0.70, Stress Management *α* of 0.69, and Group-Oriented Values *α* of 0.63. Overall, reliability indices indicated fair internal consistency for most subscales. An *α* value around 0.70 is generally considered acceptable for early-stage research and exploratory scales (Nunnally and Bernstein, 1994), while it has also been posited that constructs measuring broad, multidimensional traits may exhibit slightly lower reliability without undermining their validity (Panayides, 2013). Accordingly, the FOUND subscales demonstrate reliability levels that are appropriate given the conceptual breadth of the measured constructs. The slightly lower *α* for Group-Oriented Values likely reflects conceptual heterogeneity within this factor, consistent with expectations for complex social constructs.

#### 2.2.3 Validity testing of the FOUND

##### 2.2.3.1 Convergent validity: FOUND is distinct from mindfulness and personality trait

**Figure 1:**
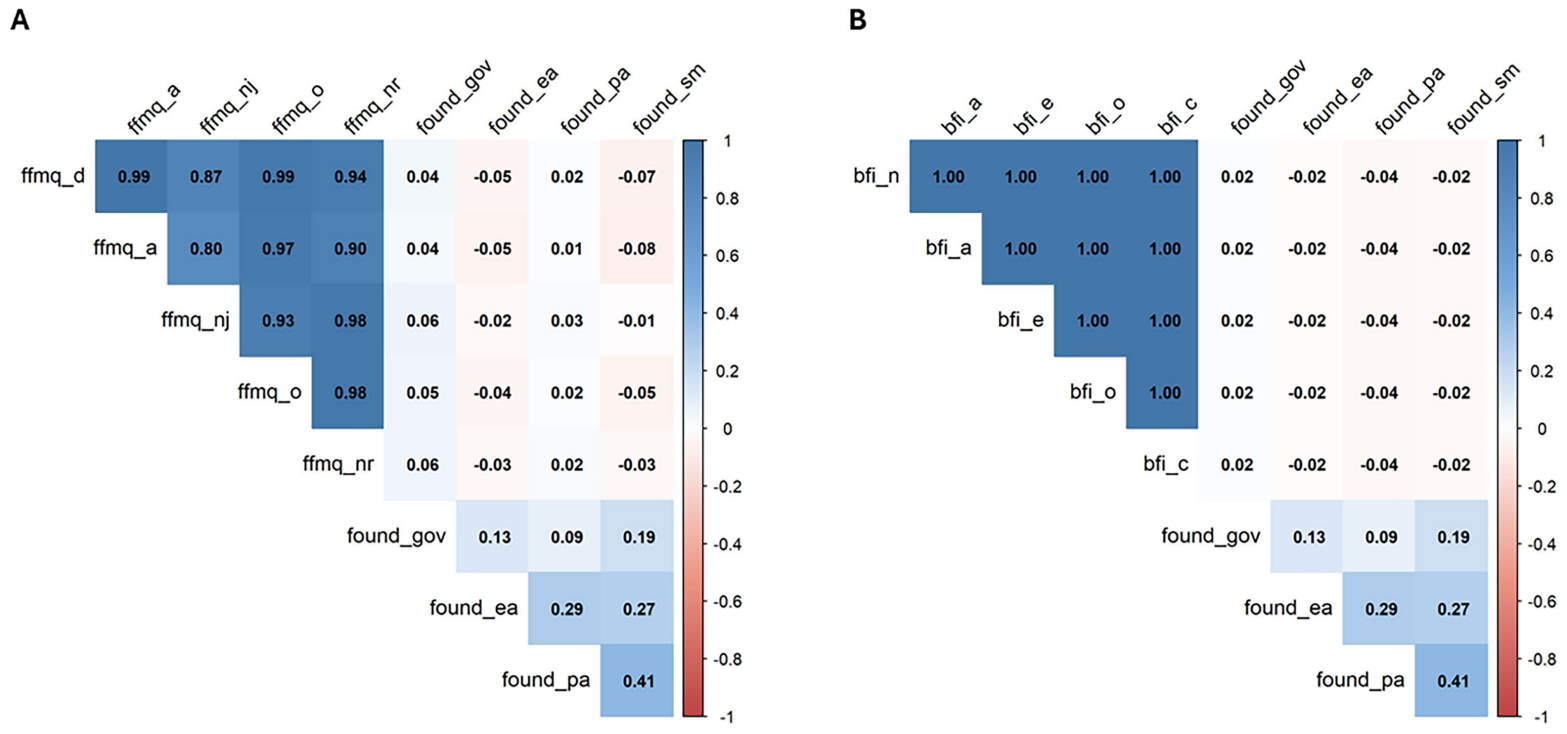
Correlation matrices for convergent validity. Color intensity reflects correlation strength (blue = positive; red = negative). Correlation coefficients are shown in the rectangles. Panel **(A)**: FOUND × FFMQ-15; Panel **(B)**: FOUND × BFI-10. FOUND – found_pa = Perception and Action; found_ea = Empathic Attitude; found_sm = Stress Management; found_gov = Group-Oriented Values. FFMQ-15 – Five Facet Mindfulness Questionnaire – 15: FFMQ-15_o = Observing; FFMQ-15_d = Describing; FFMQ-15_a = Acting with Awareness; FFMQ-15_nj = Non-judging of Inner Experience; FFMQ-15_nr = Non-reactivity to Inner Experience. BFI-10 – Big Five Inventory – 10: BFI-10_o = Openness to Experience; BFI-10_c = Conscientiousness; BFI-10_e = Extraversion; BFI-10_a = Agreeableness; BFI-10_n = Neuroticism.

The FOUND questionnaire did not exhibit any significant correlations with the factors of either the FFMQ-15 or the BFI-10 (all *p* > 0.05; Figures 1A,B).

##### 2.2.3.2 Known-groups validity: high procedural occupations show enhanced perception and action, empathy, and stress management

Within each broad occupational category, a preliminary Type III Repeated-Measures ANOVA was conducted to assess differences among subgroups (High Procedural: Creative Service, Professional Management, Technical; Low Procedural: Administrative, Field-based). These within-group analyses did not yield any significant differences, and therefore, the subgroups were aggregated into the broader High Procedural versus Low Procedural categories for subsequent analyses. Results for the subgroups are reported in Supplementary material 2 (Subgroup results: 2.1. High Procedural Occupations; 2.2. Low Procedural Occupations) and in Supplementary Figures 1, 2. Mauchly’s test of aggregated data (High Procedural vs. Low Procedural) indicated that the assumption of sphericity was not violated for either the main effect of factor (*W* = 0.944, *p* = 0.056) or the Occupation × Factor interaction (*W* = 0.944, *p* = 0.056). Greenhouse–Geisser and Huynh-Feldt corrections produced similar results.

**Figure 2:**
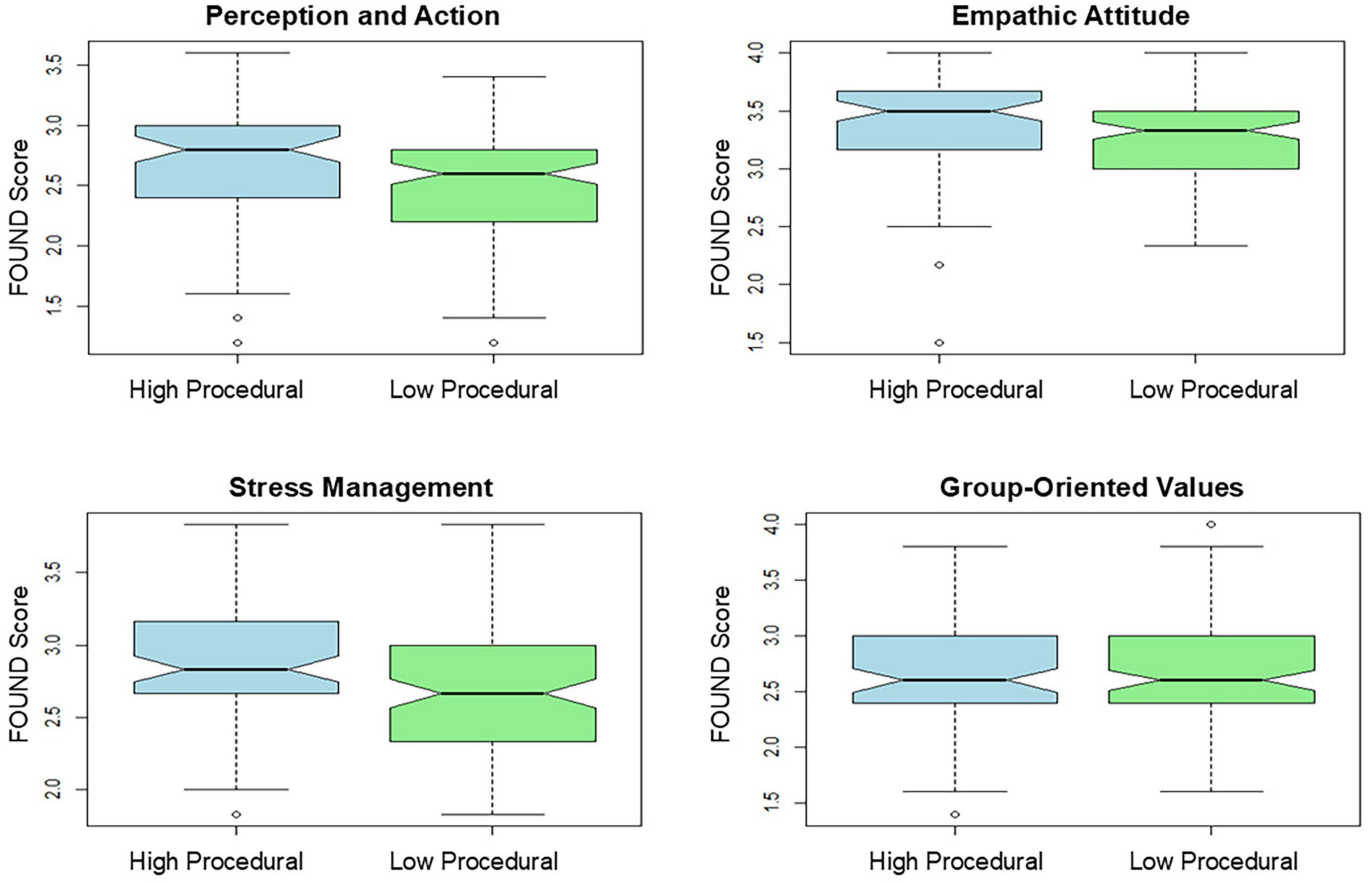
Comparison of FOUND scores between professions requiring high procedural skills and those requiring low procedural skills.

The main effect of occupation was significant (*F*(1, 187) = 8.97, *p* = 0.003, *η*^2^*ₚ* = 0.05), indicating that High Procedural participants scored slightly higher than Low Procedural participants across FOUND factors. The main effect of the factor was highly significant (*F*(3, 561) = 104.64, *p* < 0.001, *η*^2^*ₚ* = 0.36), showing that scores differ substantially across FOUND dimensions. The Occupation × Factor interaction approached significance but did not reach it (*F*(3, 561) = 2.43, *p* = 0.064, *η*^2^*ₚ* = 0.01), suggesting that differences between High Procedural and Low Procedural participants are relatively consistent across FOUND factors (Figure 2).

Post-hoc Bonferroni-corrected pairwise comparisons indicated that High Procedural participants scored significantly higher than Low Procedural participants on Perception and Action (mean difference = 0.192, *p* = 0.012, Cohen’s *d* = 0.37), Empathic Attitude (mean difference = 0.166, *p* = 0.011, *d* = 0.38), and Stress Management (mean difference = 0.181, *p* = 0.011, *d* = 0.38). No significant difference was observed for Group-Oriented Values (mean difference = −0.015, *p* = 0.827, *d* = −0.03).

These results indicate that High Procedural participants show higher scores in Perception and Action, Empathic Attitude, and Stress Management. Effect sizes are small to moderate for these dimensions (Cohen’s *d* ≈ 0.37–0.38). Partial eta-squared values showed that the largest proportion of variance is explained by differences between FOUND factors (*η*^2^*ₚ* = 0.36), while occupation accounts for a smaller portion (*η*^2^*ₚ* = 0.05) and the interaction contributes minimally (*η*^2^*ₚ* = 0.01).

## 3 Study 2

In Study 2, we performed a known-group validity to investigate putative differences in the FOUND scores between a sample of drone operators and the general population.

### 3.1 Participants

An independent sample of drone operators (age: *M* = 41 years, SD = 8.9, range = 26–57; 30 males, 4 females) was recruited voluntarily through announcements published in specialized online journals. For comparison, data from the general population sample surveyed in Study 1 were used (*n* = 300, see section 2.1.1).

All participants received a detailed protocol briefing before completing the questionnaire and were debriefed regarding the study objectives after completion.

The survey study protocol and its contents were approved by the Bioethical Committee of the University of Pisa on 28/01/2022 (n.3/2022).

#### 3.1.1 Material

The FOUND questionnaire (for a detailed description, see Study 1) was administered to participants via the Microsoft Forms platform.

#### 3.1.2 Data analysis

Data analysis focused on testing group differences. All statistical procedures were conducted using R (ver. 4.2.0) using standard packages for Welch’s t-tests and effect size computation.

##### 3.1.2.1 Known-groups validity between drone operators and the general population

To assess differences in FOUND subscale scores between individuals performing professions requiring remote manipulation (drone operators) and the general population, we conducted independent-samples t-tests. Due to the substantial inequality in sample sizes between the two groups (*n* = 34 drone operators vs. *n* = 300 general population) and the possibility of unequal variances, Welch’s t-test was used, as it provides a robust alternative to the standard Student’s t-test under these conditions. The FOUND questionnaire factors included in the analysis were Perception and Action, Empathic Attitude, Stress Management, and Group-Oriented Values. For each comparison, Hedges’ *g* was calculated as a measure of effect size, providing a bias-corrected estimate appropriate for small and unequal sample sizes. This approach allowed us to quantify the magnitude of differences between drone operators and the general population across each of the four FOUND subscales.

### 3.2 Results

#### 3.2.1 Drone operators exhibit enhanced perception and action, higher group-oriented values, and reduced stress management compared to the general population

**Figure 3:**
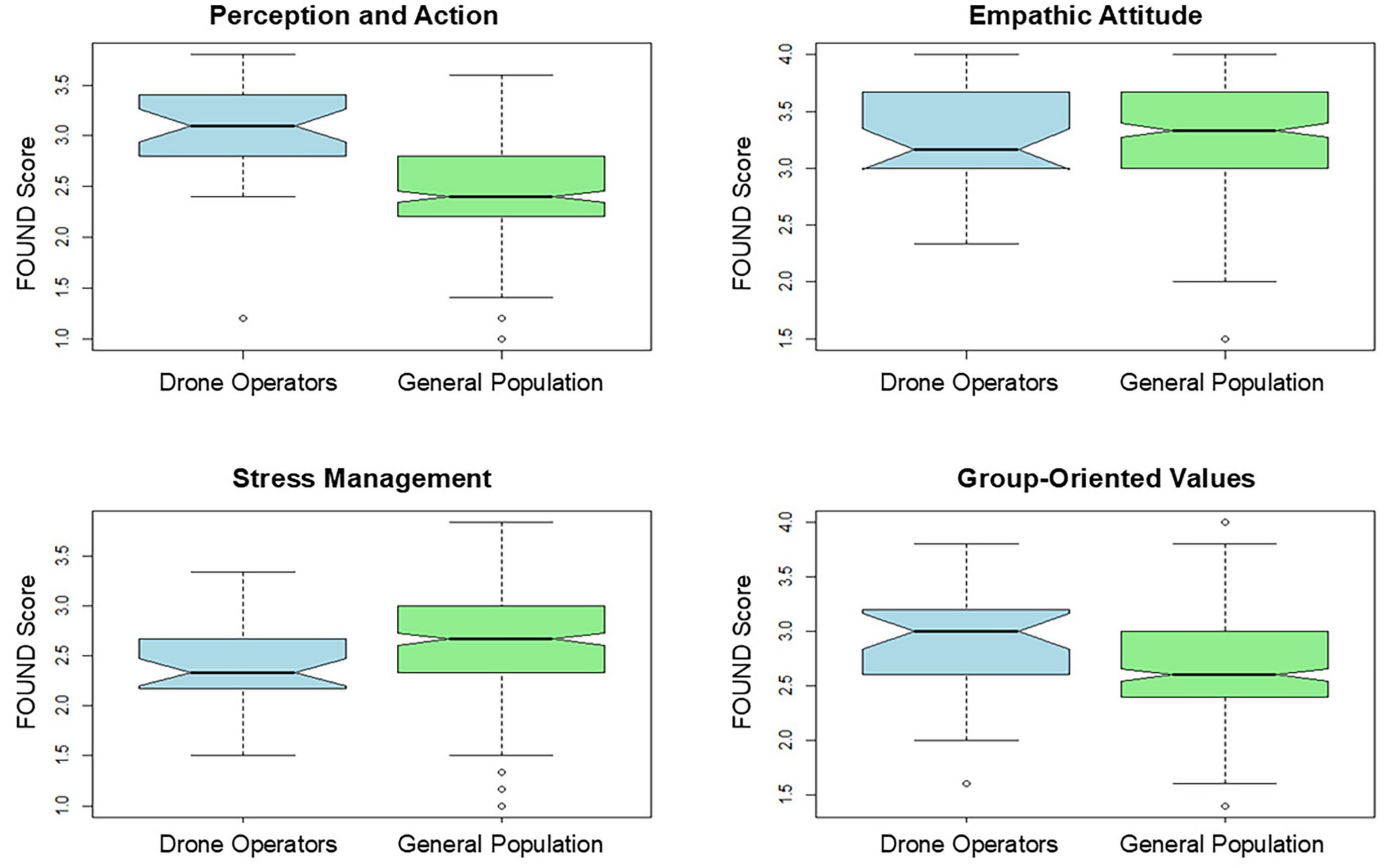
Comparison of FOUND scores between drone operators and the general population.

Participants in professions requiring remote manipulation (*n* = 34) were compared with the general population (*n* = 300) on the FOUND subscales using Welch’s t-tests. The results showed that the remote manipulation group scored significantly higher on Perception and Action (*t* = 6.45, df = 43.03, *p* < 0.001, Hedges’ *g* = 0.998), significantly lower on Stress Management (*t* = −4.10, df = 49.85, *p* = 0.00015, *g* = 0.55), and higher on Group-Oriented Values (*t* = 2.68, df = 41.63, *p* = 0.011, *g* = 0.36). No significant difference was observed for Empathic Attitude (*t* = 0.22, df = 42.42, *p* = 0.83, *g* = 0.03) (Figure 3). The Hedges’ *g* values indicate that the difference in Perception and Action represents a large effect, the difference in Stress Management represents a medium effect, and the difference in Group-Oriented Values represents a small-to-medium effect. Differences in Empathic Attitude are negligible.

## 4 Discussion

According to our initial hypothesis, the FOUND questionnaire encompasses four distinct factors: the perceptual and action domain, the empathic attitude, the ability to cope with stress, and the adherence to group-oriented dynamics. However, the content of each factor represents more specific aspects compared to what was initially hypothesized. For example, the cognitive and behavioral domains have been circumscribed to the perception of external and internal cues and their awareness, thus encompassing perceptual knowledge and action. In some items, individuals are required to express their agreement regarding their ability to integrate non-bodily objects, the perception of their body as adaptable and capable of various physical extensions: this implies a high degree of bodily awareness and control, which is essential for skilled motor activities and the cognitive ability to manipulate one’s body in space. Importantly, these abilities have been assessed and used in questionnaires investigating state characteristics, and not to detect individual stable traits (Gonzalez-Franco and Peck, 2018; Toet et al., 2020).

Although initially conceptualized as value-based—including ethical and motivational orientations—this domain emerged as primarily reflecting Group-Oriented Values and external expectations. Specifically, compliance involves adjusting one’s behavior in response to group norms or expectations (Jhangiani and Tarry, 2022). According to research on social influence, conformity and compliance are driven by motivations to maintain accurate perceptions of reality, preserve meaningful social relationships, and sustain a positive self-concept, often through subtle and indirect processes outside of conscious awareness (Cialdini and Goldstein, 2004). This may be helpful in mediated environments when the perceived distance between team members could exert a detrimental influence on individual decision-making. For example, Group-Oriented Values and expectations may improve coordination between individuals in long-distance team working contexts in which members refer to a team leader.

The Empathic Attitude factor aligns with our initial hypothesis, capturing the expected features of the socio-emotional domain. In this line, assessing the individual’s ability to empathize with others and feel an emotional connection is a pivotal factor in reducing social distance among users. Actually, the ability to share emotional content (and specifically appreciation toward others) is a bedrock for interpersonal closeness both in face-to-face and online interaction (Balters et al., 2023).

The Stress Management factor seems especially relevant for evaluating how individuals regulate stress and implement coping strategies. Contrary to our hypothesis, this factor examines only individual strategies to face challenges, with the prevalence of cognitive ones (such as “Even when my workload is high, I manage not to get disturbed during my leisure time.”), without exploring the more general organic and functional individuals’ domain (such as sleep habits, eating patterns, and daily routines). The ability to perceive, cope with, and mitigate stressors is a crucial construct for understanding how individuals navigate challenging situations and maintain their well-being at the same time, such as in work settings involving mediated technology, in which additional stressors may occur (higher workload, digital, and technical difficulties) (Tarafdar et al., 2007; Ayyagari et al., 2011).

Contrary to our initial hypothesis of convergent validity, our questionnaire did not show significant correlations with other questionnaires assessing personality and stable traits (BFI-10, FFMQ-15). This lack of convergence may be attributed to the unique variation in stable individual functioning, which may evaluate distinct and innovative stable characteristics. As an example, the lack of convergence between Stress Management and Neuroticism (the opposite pole of Emotional Stability), could be explained by the fact that individuals with neurotic traits may have developed effective strategies to face challenges over time: neuroticism is more related to the individual’ experience of stressors as threatening factors, not considering the ability to cope with them (Thompson, 2008).

At the same time, the lack of convergent validity between Stress Management and Non-Reactivity a core aspect of mindfulness, referring to the process of observing thoughts, emotions, and bodily sensations without reacting to them (Baer et al., 2012) could be explained by the slightly different focus of the investigation: for example, Non-Reactivity might be assessed by the degree to which an individual can observe stress-inducing thoughts without reacting to them, whereas Stress Management might be evaluated based on the effectiveness with which an individual employs techniques to mitigate stress: in other words, it is possible to possess the ability to observe stressors without reactions, but not that of actively coping with them. Thus, the FOUND Stress Management factor captures practical coping strategies in task-specific contexts.

Another example is provided by the Perception and Action domain of the FOUND questionnaire, which did not show significant correlation with the Acting with Awareness or Observing facets of the FFMQ-15. This lack of convergence can be explained by the specificity of the FOUND domain: while FFMQ-15 facets measure general attentional control and awareness of internal and external experiences (Baer et al., 2012), Perception and Action captures stable, task-specific traits primarily related to body–tool integration, multisensory processing, and the coordination of complex motor actions in mediated environments such as teleoperation or robotic surgery. Thus, the lack of convergence highlights the novelty of the FOUND questionnaire, as it assesses enduring perceptual-motor traits that are not captured by existing mindfulness measures.

Similarly, the Empathic Attitude in FOUND did not show a significant correlation with Agreeableness from the BFI-10. In fact, the BFI-10 evaluates general tendencies to be trusting and not to identify flaws in others (Rammstedt and John, 2007), while Empathic Attitude in FOUND captures task- and context-specific prosocial tendencies, including the ability to recognize, respond to, and support others, thus primarily reflecting stable behavioral dispositions in collaborative rather than broad dispositional warmth.

Ultimately, the Group-Oriented Values factor belonging to the FOUND questionnaire did not show significant correlations with the BFI-10 Agreeableness or the Acting with Awareness and Observing facets of the FFMQ-15. This lack of convergence can be explained by the specificity of the FOUND construct. Specifically, while Agreeableness detects general tendencies toward trusting (Rammstedt and John, 2007), and the FFMQ-15 facets assess individual attentional and present-moment awareness (Baer et al., 2012), Group-Oriented Values reflect context-specific traits related to adherence to group norms, structured teamwork, social coordination, and collective responsibility.

In this vein, the lack of convergent validity can be interpreted not as a limitation, but rather as evidence that FOUND taps novel, task-relevant constructs that are not detected by existing trait scales. This evidence may support the scale’s innovative contribution to identifying stable traits that specifically underpin performance in mediated and remote environments.

By comparing professions with high versus low procedural demands, we observed significant differences in the FOUND factors scores. Specifically, professions requiring greater procedural skills exhibited higher scores in Perception and Action, Empathic Attitudes, and Stress Management compared to those with lower procedural demands.

The higher scores in Perception and Action, Empathic Attitudes, and Stress Management among individuals involved in professions with high procedural ability may be attributed to the specific demands and nature of these roles. Most of these professions require the advanced, step-by-step application of perceptual and motor coordination, as well as the ability to solve problems or apply strategies that rely on non-declarative memory systems (VanLehn, 1996; Rosenbaum et al., 2001; Ackerman, 2007). These kinds of skills mainly take advantage of experience and repetition. To better summarize these characteristics, three main stages have been defined: the cognitive stage (involving the setting of goals and the planning of actions using explicit knowledge), the associative stage (involving the refinement of techniques and the exploration of possible variations), and the automatic stage [involving the automation of skills and the reduction in the time required for their execution (Fitts and Posner, 1967)]. These stages are inextricably linked to individuals’ experiences, achieved through numerous repetitions (Cesari et al., 2024). In this regard, we can assume that professions with higher procedural demands require a period to consolidate the so-called “know-how” experience through different stages, and this advantage could explain the higher score in the perceptual and action domain. Conversely, it is also possible that traits such as perceptual and action abilities might influence the professional choice of occupations with high procedural demands.

Similarly, the higher Stress Management score in individuals performing professions requiring higher procedural abilities might be explained by the fact that stress can induce memory shifts (from explicit to implicit) and, from an evolutionary perspective, enable the human brain to extract probabilistic information embedded in the environment in stressful situations more rapidly. This stress-related enhancement provides an advantage for the individual to effectively manage the situation (Fournier et al., 2017; Wirz et al., 2018; Tóth-Fáber et al., 2020).

In parallel, the higher empathic attitude score in individuals performing professions requiring higher procedural abilities might be explained by the automaticity of caring or empathic attitudes (Brown et al., 2011), which can be prompted by experience in performing procedural skills.

Finally, our results show that individuals who perform remote operations, compared to the general population, display higher scores in Perception and Actions and Group-Oriented Values scores, but lower scores in Stress Management ability. The differences in Perception and Action are expected since people using remote manipulation are more likely to perceive remote controllers as a natural extension of the body (Toet et al., 2020), and in parallel, the better spatial orientation could be ascribed to their extensive training and operational experience, which helps these individuals to develop higher spatial orientation skills (Cooke, 2006).

Higher FOUND scores on Group-Oriented Values among remote operators can be understood as the outcome of professional environments that emphasize structured training, high-risk operations (e.g., military drone tasks), and continuous monitoring (Salas and Cannon-Bowers, 2001). These conditions may reinforce adherence to shared norms, team coordination, and collective responsibility, illustrating how professional contexts can cultivate group-oriented behaviors.

Additionally, the lower scores in Stress Management among individuals engaged in remote operations may be attributed to the diminished control inherent in remote settings. This diminished control can be attributed to several factors, including limited autonomy, dependency on technology, and physical detachment from the operational settings (Sam et al., 2024). Furthermore, teleoperation environments, which are typified by their unpredictability and lack of certainty regarding future events, present considerable challenges that may additionally impair individuals’ stress management capabilities (Toet et al., 2020).

## 5 Limitations

The current work, although exploratory, presents several limitations that should be carefully considered when interpreting the results. The study’s use of a relatively modest sample for the CFA raises concerns about whether the data are sufficient to robustly validate a complex four-factor 22-item model, thus precluding the generalizability of our findings. This limitation suggests that the CFA results should be viewed as preliminary. In addition, the participants’ sample involved in Study 2—particularly those with remote operation experience—was relatively limited. This smaller group size, while informative, precludes robust conclusions and underscores the need for future studies involving larger and more diverse samples. A further limitation concerns gender distribution in Study 2. Specifically, the group of remote operators was predominantly male (females, *n* = 4), reflecting existing gender imbalances in certain technical professions. This also limits the exploration of potential gender-related differences in trait expression. Achieving a more balanced gender representation in future research would help determine whether the FOUND factors differ meaningfully across gender lines.

Another important consideration concerns the cultural context of enrolled individuals. Participants in this study were exclusively Italian, which may limit the generalizability of the findings to other cultural or linguistic groups. Cultural norms and values can influence both the interpretation of questionnaire items and the expression of stable traits in professional settings. Cross-cultural validation of the FOUND questionnaire is therefore crucial to ensure its applicability beyond the Italian cultural context.

Another limitation is related to the Perception and Action factor of the FOUND questionnaire. Specifically, while designed to capture stable perceptual-motor traits, it may also be influenced by prior experience and procedural skill. This overlap introduces some ambiguity, as higher scores could reflect both enduring traits and acquired expertise. Future research should aim to disentangle these contributions, for example, through longitudinal studies or by controlling for participants’ experience levels.

Another issue to consider is the lack of convergent validity between the FOUND questionnaire and related instruments such as the BFI-10 and FFMQ-15. While the absence of significant correlations may suggest that FOUND captures novel or distinct aspects of individual differences, alternative explanations should be acknowledged. Low correlations might also reflect conceptual mismatches between constructs, measurement error, or insufficient statistical power. Given that some degree of overlap with existing personality measures was anticipated, the divergence raises interpretive questions that warrant further investigation. This limitation underscores the importance of future studies aimed at clarifying the conceptual space of the FOUND scale and its relationship to established trait frameworks.

Another limitation is related to the heterogeneity of the subgroups within the High Procedural and Low Procedural categories. Although comparisons within these subgroups revealed no significant differences for FOUND constructs, the broad occupational categorization could mask subtle distinctions in procedural or trait-related abilities. Aggregating professions into high versus low procedural groups simplifies interpretation but may obscure nuanced distinctions among specific occupations. Finally, a crucial limitation is that the present study does not test whether FOUND scores actually predict performance outcomes in remote tasks. As such, claims regarding the practical applicability of the questionnaire remain tentative. Moreover, the study relies solely on self-report measures, which may be subject to biases such as social desirability or misinterpretation of item content. Including behavioral or physiological assessments in future research could provide additional evidence, allowing for the evaluation of predictive and incremental validity of the FOUND questionnaire.

## 6 Conclusion

This study introduces and validates the FOUND questionnaire, conceived for assessing stable psychological traits that can affect the use of new media and teleoperations. From the questionnaire validation process, we obtained four factors: (i) Perception and Action, (ii) Empathic Attitude, (iii) Stress Management, and (iv) Group-Oriented Values. These factors cover multiple domains of individuals’ functioning and may be used to assess stable subjective traits in professional settings by synthesizing key constructs from perceptual, motor, cognitive, social, and emotional domains into a single questionnaire. FOUND can discriminate (known-groups validity) between individuals performing different professions (professions with high versus low procedural demand; individuals in remote manipulation settings versus the general population), thus paving the way for the description of individuals who confront the demands of remote settings to achieve optimal performance. The use of the FOUND questionnaire in remote operation settings, such as robotic surgery, and in environments that utilize online platforms, such as telemedicine, education, and emergency response, provides an opportunity to assess individuals’ personality attitudes while accounting for performance indices. Furthermore, understanding stable traits in these contexts could facilitate the development of personalized interventions informed by individual stable characteristics.

## Data Availability

The raw data supporting the conclusions of this article will be made available by the authors without undue reservation.

## References

[ref1] Ackerman P. L. (2007). New developments in understanding skilled performance. Curr. Dir. Psychol. Sci. 16, 235–239. doi: 10.1111/j.1467-8721.2007.00511.x

[ref2] Anderson J. R. (1976). Language, memory, and thought. Oxford, England: Lawrence Erlbaum.

[ref3] Ashton M. C. Lee K. (2007). Empirical, theoretical, and practical advantages of the HEXACO model of personality structure. Personal. Soc. Psychol. Rev. 11, 150–166. doi: 10.1177/1088868306294907, PMID: 18453460

[ref4] Ayyagari R. Grover V. Purvis R. (2011). Technostress: technological antecedents and implications. MIS Q. 35, 831–858. doi: 10.2307/41409963

[ref5] Baer R. A. Carmody J. Hunsinger M. (2012). Weekly change in mindfulness and perceived stress in a mindfulness-based stress reduction program. J. Clin. Psychol. 68, 755–765. doi: 10.1002/jclp.21865, PMID: 22623334

[ref6] Balters S. Miller J. G. Li R. Hawthorne G. Reiss A. L. (2023). Virtual (zoom) interactions alter conversational behavior and interbrain coherence. J. Neurosci. 43, 2568–2578. doi: 10.1523/JNEUROSCI.1401-22.2023, PMID: 36868852 PMC10082458

[ref7] Boateng G. O. Neilands T. B. Frongillo E. A. Melgar-Quiñonez H. R. Young S. L. (2018). Best practices for developing and validating scales for health, social, and behavioral research: a primer. Front. Public Health 6:149. doi: 10.3389/fpubh.2018.00149, PMID: 29942800 PMC6004510

[ref8] Botvinick M. Cohen J. (1998). Rubber hands ‘feel’ touch that eyes see. Nature 391:756. doi: 10.1038/35784 9486643

[ref9] Brown W. S. Garrels S. R. Reimer K. S. (2011). Mimesis and compassion in care for people with disabilities. J. Relig. Disabil. Health 15, 377–394. doi: 10.1080/15228967.2011.620383

[ref10] Brunyé T. T. Goring S. A. Cantelon J. A. Eddy M. D. Elkin-Frankston S. Elmore W. R. . (2024). Trait-level predictors of human performance outcomes in personnel engaged in stressful laboratory and field tasks. Front. Psychol. 15:1449200. doi: 10.3389/fpsyg.2024.1449200, PMID: 39315045 PMC11418282

[ref11] Burin D. Pignolo C. Ales F. Giromini L. Pyasik M. Ghirardello D. . (2019). Relationships between personality features and the rubber hand illusion: an exploratory study. Front. Psychol. 10:2762. doi: 10.3389/fpsyg.2019.02762, PMID: 31920815 PMC6914866

[ref12] Cattell R. B. (1946). Description and measurement of personality. Oxford, England: World Book Company.

[ref13] Cegarra-Navarro J.-G. Wensley A. Jimenez-Jimenez D. Sotos-Villarejo A. (2017). Linking procedural memory with organizational learning through knowledge corridors. J. Knowl. Manag. 21, 1503–1522. doi: 10.1108/JKM-01-2017-0018

[ref14] Cesari V. D’Aversa S. Piarulli A. Melfi F. Gemignani A. Menicucci D. (2024). Sense of agency and skills learning in virtual-mediated environment: a systematic review. Brain Sci. 14:350. doi: 10.3390/brainsci14040350, PMID: 38672002 PMC11048251

[ref15] Cesari V. Galgani B. Gemignani A. Menicucci D. (2021). Enhancing qualities of consciousness during online learning via multisensory interactions. Behav. Sci. 11:57. doi: 10.3390/bs11050057, PMID: 33919379 PMC8143304

[ref16] Cialdini R. B. Goldstein N. J. (2004). Social influence: compliance and conformity. Annu. Rev. Psychol. 55, 591–621. doi: 10.1146/annurev.psych.55.090902.142015, PMID: 14744228

[ref17] Cohen S. Kamarck T. Mermelstein R. (1983). A global measure of perceived stress. J. Health Soc. Behav. 24, 385–396. doi: 10.2307/2136404 6668417

[ref18] Cooke N. J. (2006). Human factors of remotely operated vehicles. Proc. Hum. Factors Ergon. Soc. Annu. Meet. 50, 166–169. doi: 10.1177/154193120605000135

[ref19] Costantini G. Saraulli D. Perugini M. (2020). Uncovering the motivational core of traits: the case of conscientiousness. Eur. J. Personal. 34, 1073–1094. doi: 10.1002/per.2237

[ref20] Dissaux T. Jancart S. (2023). “The impact of procedural knowledge retrieval on the architectural design process in parametric design environments” in Design computing and cognition’22. ed. Gero J. S. (Cham: Springer International Publishing), 681–697.

[ref21] Enayati N. De Momi E. Ferrigno G. (2016). Haptics in robot-assisted surgery: challenges and benefits. IEEE Rev. Biomed. Eng. 9, 49–65. doi: 10.1109/RBME.2016.2538080, PMID: 26960228

[ref22] Fitts P. M. Posner M. I. (1967). Human performance. Oxford, England: Brooks/Cole.

[ref23] Fournier M. d’Arripe-Longueville F. Radel R. (2017). Effects of psychosocial stress on the goal-directed and habit memory systems during learning and later execution. Psychoneuroendocrinology 77, 275–283. doi: 10.1016/j.psyneuen.2016.12.008, PMID: 28131067

[ref24] Goldberg L. R. (1990). An alternative “description of personality”: the big-five factor structure. J. Pers. Soc. Psychol. 59, 1216–1229. doi: 10.1037//0022-3514.59.6.1216, PMID: 2283588

[ref25] Goldberg L. R. (1993). The structure of phenotypic personality traits. Am. Psychol. 48, 26–34. doi: 10.1037//0003-066x.48.1.26, PMID: 8427480

[ref26] Gonzalez-Franco M. Peck T. C. (2018). Avatar embodiment. Towards a standardized questionnaire. Front. Robot. AI 5:74. doi: 10.3389/frobt.2018.00074, PMID: 33500953 PMC7805666

[ref27] Green P. Edwards E. J. Tower M. (2022). Core procedural skills competencies and the maintenance of procedural skills for medical students: a Delphi study. BMC Med. Educ. 22:259. doi: 10.1186/s12909-022-03323-9, PMID: 35397566 PMC8994896

[ref28] Groza M. D. Groza M. P. (2018). Salesperson regulatory knowledge and sales performance. J. Bus. Res. 89, 37–46. doi: 10.1016/j.jbusres.2018.04.005

[ref29] Guru K. A. Shafiei S. B. Khan A. Hussein A. A. Sharif M. Esfahani E. T. (2015). Understanding cognitive performance during robot-assisted surgery. Urology 86, 751–757. doi: 10.1016/j.urology.2015.07.028, PMID: 26255037

[ref30] Hagen M. E. Meehan J. J. Inan I. Morel P. (2008). Visual clues act as a substitute for haptic feedback in robotic surgery. Surg. Endosc. 22, 1505–1508. doi: 10.1007/s00464-007-9683-0, PMID: 18071811

[ref31] Hayton J. C. Allen D. G. Scarpello V. (2004). Factor retention decisions in exploratory factor analysis: a tutorial on parallel analysis. Organ. Res. Methods 7, 191–205. doi: 10.1177/1094428104263675

[ref32] Jayawickreme E. Zachry C. E. (2020). “Traits and dynamic processes” in The Cambridge handbook of personality psychology. eds. Matthews G. Corr P. J. (Cambridge: Cambridge University Press), 352–363.

[ref33] Jhangiani D. R. Tarry D. H. (2022). Principles of social psychology - 1st international H5P edition. BCcampus. Available online at: https://opentextbc.ca/socialpsychology/ (Accessed January 8, 2025).

[ref34] Johnsen B. H. Nilsen A. A. Hystad S. W. Grytting E. Ronge J. L. Rostad S. . (2024). Selection of Norwegian police drone operators: an evaluation of selected cognitive tests from “the Vienna test system.”. Police Pract. Res. 25, 38–52. doi: 10.1080/15614263.2023.2179052

[ref35] Kilteni K. Groten R. Slater M. (2012). The sense of embodiment in virtual reality. Presence Teleoper. Virtual Environ. 21, 373–387. doi: 10.1162/PRES_a_00124

[ref36] Knowlton B. J. Siegel A. L. M. Moody T. D. (2017). “Procedural learning in humans,” in Memory Systems, Vol. 3 of Learning and memory: A comprehensive. ed. Eichenbaum H.. 2nd edn. Oxford: Elsevier, 295–312.

[ref37] Li C.-H. (2016). Confirmatory factor analysis with ordinal data: comparing robust maximum likelihood and diagonally weighted least squares. Behav. Res. Methods 48, 936–949. doi: 10.3758/s13428-015-0619-7, PMID: 26174714

[ref38] Lynn M. R. (1986). Determination and quantification of content validity. Nurs. Res. 35, 382–385. doi: 10.1097/00006199-198611000-00017, PMID: 3640358

[ref39] Magdalon E. C. Michaelsen S. M. Quevedo A. A. Levin M. F. (2011). Comparison of grasping movements made by healthy subjects in a 3-dimensional immersive virtual versus physical environment. Acta Psychol. 138, 126–134. doi: 10.1016/j.actpsy.2011.05.015, PMID: 21684505

[ref40] Martin J. (1987). Double-coding: a key to knowledge utilization and generation in the instruction and learning of skills. Instrum. Sci. 16, 47–58. doi: 10.1007/BF00120005

[ref41] McCrae R. R. Costa P. T. (1987). Validation of the five-factor model of personality across instruments and observers. J. Pers. Soc. Psychol. 52, 81–90. doi: 10.1037//0022-3514.52.1.81, PMID: 3820081

[ref42] McCrae R. R. Costa P. T. Jr. (1999). “A five-factor theory of personality” in Handbook of personality: theory and research. 2nd ed (New York, NY, US: Guilford Press), 139–153.

[ref43] Menicucci D. Piarulli A. Laurino M. Zaccaro A. Agrimi J. Gemignani A. (2020). Sleep slow oscillations favour local cortical plasticity underlying the consolidation of reinforced procedural learning in human sleep. J. Sleep Res. 29:e13117. doi: 10.1111/jsr.13117, PMID: 32592318

[ref44] Muller T. Subrin K. Joncheray D. Billon A. Garnier S. (2022). Transparency analysis of a passive heavy load comanipulation arm. IEEE Trans. Hum.-Mach. Syst. 52, 918–927. doi: 10.1109/THMS.2022.3156887

[ref45] Newell A. Simon H. A. (1972). Human problem solving. Oxford, England: Prentice-Hall.

[ref46] Nunnally J. C. Bernstein I. H. (1994). Psychometric theory. New York, NY: McGraw-Hill Companies, Incorporated.

[ref47] Ottley A. (2020). “Cognitive traits that matter” in Adaptive and personalized visualization. ed. Ottley A. (Cham: Springer International Publishing), 9–15.

[ref48] Palan S. Schitter C. (2018). Prolific.ac—a subject pool for online experiments. J. Behav. Exp. Finance 17, 22–27. doi: 10.1016/j.jbef.2017.12.004

[ref49] Panayides P. (2013). Coefficient alpha: interpret with caution. Eur. J. Psychol. 9, 687–696. doi: 10.5964/ejop.v9i4.653

[ref50] Perepelkina O. Boboleva M. Arina G. Nikolaeva V. (2017). Higher emotional intelligence is associated with a stronger rubber hand illusion. Multisens. Res. 30, 615–637. doi: 10.1163/22134808-00002577

[ref51] Qin K. Zhang Y. Zhang J. (2022). “A study on the influence of personality on the performance of teleoperation tasks in different situations” in Engineering psychology and cognitive ergonomics: 19th international conference, EPCE 2022, held as part of the 24th HCI international conference, HCII 2022, virtual event, June 26 – July 1, 2022, proceedings (Berlin, Heidelberg: Springer-Verlag), 212–224.

[ref52] Rammstedt B. John O. P. (2007). Measuring personality in one minute or less: a 10-item short version of the big five inventory in English and German. J. Res. Pers. 41, 203–212. doi: 10.1016/j.jrp.2006.02.001

[ref53] Revelle W. (2015). psych: Procedures for psychological, psychometric, and personality research. Evanston, Illinois: Northwestern University.

[ref54] Ringwald W. R. Napolitano C. M. Sewell M. N. Soto C. J. Yoon H. J. Wright A. G. (2025). More skill than trait, or more trait than skill? Relations of (mis)matches between personality traits and social, emotional, and behavioral skills with adolescent outcomes. Eur. J. Personal., 08902070241309960. doi: 10.1177/08902070241309960

[ref55] Roccas S. Sagiv L. Schwartz S. H. Knafo A. (2002). The big five personality factors and personal values. Personal. Soc. Psychol. Bull. 28, 789–801. doi: 10.1177/0146167202289008

[ref56] Rosenbaum D. A. Carlson R. A. Gilmore R. O. (2001). Acquisition of intellectual and perceptual-motor skills. Annu. Rev. Psychol. 52, 453–470. doi: 10.1146/annurev.psych.52.1.453, PMID: 11148313

[ref57] Rosseel Y. (2012). lavaan: an R package for structural equation modeling. J. Stat. Softw. 48, 1–36. doi: 10.18637/jss.v048.i02

[ref58] Salas E. Cannon-Bowers J. A. (2001). The science of training: a decade of progress. Annu. Rev. Psychol. 52, 471–499. doi: 10.1146/annurev.psych.52.1.471, PMID: 11148314

[ref59] Sam Y. T. Hedlund-Botti E. Natarajan M. Heard J. Gombolay M. (2024). The impact of stress and workload on human performance in robot teleoperation tasks. IEEE Trans. Robot. 40, 4725–4744. doi: 10.1109/TRO.2024.3484630

[ref60] Satorra A. Bentler P. M. (1994). “Corrections to test statistics and standard errors in covariance structure analysis” in Latent variables analysis: Applications for developmental research (Thousand Oaks, CA, US: Sage Publications, Inc), 399–419.

[ref61] Smith E. A. (2001). The role of tacit and explicit knowledge in the workplace. J. Knowl. Manag. 5, 311–321. doi: 10.1108/13673270110411733

[ref62] Tabachnick B. G. Fidell L. S. (2019). Using multivariate statistics. Seventh Edn. New York, NY: Pearson.

[ref63] Tarafdar M. Tu Q. Ragu-Nathan B. S. Ragu-Nathan T. S. (2007). The impact of technostress on role stress and productivity. J. Manage. Inf. Syst. 24, 301–328. doi: 10.2753/MIS0742-1222240109

[ref64] Thompson E. R. (2008). Development and validation of an international English big-five mini-markers. Pers. Individ. Differ. 45, 542–548. doi: 10.1016/j.paid.2008.06.013

[ref65] Toet A. Kuling I. A. Krom B. N. van Erp J. B. F. (2020). Toward enhanced teleoperation through embodiment. Front. Robot. AI 7:14. doi: 10.3389/frobt.2020.00014, PMID: 33501183 PMC7805894

[ref66] Tóth-Fáber E. Janacsek K. Szőllősi Á. Kéri S. Németh D. (2020). Procedural learning under stress: boosted statistical learning but unaffected sequence learning. 2020.05.13.092726. doi: 10.1101/2020.05.13.092726

[ref67] VanLehn K. (1996). Cognitive skill acquisition. Annu. Rev. Psychol. 47, 513–539. doi: 10.1146/annurev.psych.47.1.513, PMID: 15012487

[ref68] Voronchuk I. Starineca O. (2014). Knowledge management and possibilities of professional development in public sector. Eur. Integr. Stud. 168–179. doi: 10.5755/j01.eis.0.8.6844

[ref69] Wang Y. Tree J. E. F. Walker M. Neff M. (2016). Assessing the impact of hand motion on virtual character personality. ACM Trans. Appl. Percept. 13, 1–23. doi: 10.1145/2874357

[ref70] Wirz L. Bogdanov M. Schwabe L. (2018). Habits under stress: mechanistic insights across different types of learning. Curr. Opin. Behav. Sci. 20, 9–16. doi: 10.1016/j.cobeha.2017.08.009

[ref71] Yeh S.-L. Lane T. J. Chang A.-Y. Chien S.-E. (2017). Switching to the rubber hand. Front. Psychol. 8:2172. doi: 10.3389/fpsyg.2017.02172, PMID: 29312048 PMC5732964

