# Correction: The FOUND questionnaire: identifying stable traits associated with success in remote operations—an exploratory study

**DOI:** 10.3389/fnsys.2025.1777499

**Published:** 2026-01-13

**Authors:** Valentina Cesari, Enrico Cipriani, Giorgia Papini, Andrea Piarulli, Angelo Gemignani, Danilo Menicucci

**Affiliations:** Department of Surgical, Medical and Molecular Pathology and Critical Care Medicine, University of Pisa, Pisa, Italy

**Keywords:** personality, traits, telemanipulation, embodiment, skills learning, dexterity

The original version of this article has been updated.

In the published article, a funding source was erroneously omitted from the Funding statement. The following sentence should have been added: “Funding was also provided through the European Union – Next Generation EU, in the context of the National Recovery and Resilience Plan, THE – Tuscany Health Ecosystem, ECS00000017, CUP I53C22000780001 to AP.” The corrected Funding statement appears below.

