# Supplementary material for: The FOUND questionnaire: identifying stable traits associated with success in remote operations—an exploratory study
Source: Front Syst Neurosci. 2025 Nov 6;19:1676412. doi: 10.3389/fnsys.2025.1676412 (PMC12631305; doi:10.3389/fnsys.2025.1676412)
Supplement: Supplementary file 1 [file Table_1.DOCX]

Supplementary Material

# FOUND QUESTIONNAIRE

- 1. **FOUND questionnaire – Italian**

Istruzioni: Le seguenti affermazioni si riferiscono a modi di agire, pensare e sentire propri della vita quotidiana. Attribuisca il punteggio che più si avvicina alla sua esperienza sulla base dell'affermazione presentata.

| 1  Fortemente in disaccordo | 2  In disaccordo | 3  D’accordo | 4  Fortemente  d’accordo |
| --- | --- | --- | --- |

1. È essenziale comportarsi correttamente per evitare di mostrare condotte giudicate come sbagliate.
2. Mi ritengo più abile di altre persone quando devo orientarmi nello spazio.
3. Dopo una notte di sonno, mi sento pieno/a di forza per affrontare la giornata.
4. Saper ascoltare le ragioni degli altri è un valore aggiunto alla conoscenza.
5. Penso che ogni persona debba essere meritevole di cura e amore.
6. Percepisco il mio corpo come malleabile ed estendibile.
7. Provo piacere nel far star bene le persone.
8. **(R)** Preferisco delegare gli altri quando devo monitorare azioni che coinvolgono altre persone.
9. **(R)** Se sono in uno spazio sconosciuto e senza riferimenti, ho difficoltà ad orientarmi.
10. In un gruppo, è necessario che ci sia una guida che indirizzi il lavoro di tutti.
11. Penso che gli interessi personali debbano essere messi da parte per il bene di un gruppo.
12. Essere proattivi verso coloro che soffrono è un pilastro fondamentale della mia vita.
13. Anche quando il mio carico di lavoro è elevato, riesco a non farmi perturbare nei momenti di svago.
14. Se utilizzo un oggetto, questo diventa un’estensione naturale del mio corpo.
15. **(R)** Trovo difficoltà ad immergermi nelle attività che devo svolgere.
16. Essere dediti a qualcosa significa dedicarsi completamente, senza concedersi altro.
17. Penso che il lavoro di squadra sia gratificante.
18. Penso che l’obbedienza sia fondamentale nella vita.
19. Quando mi trovo ad affrontare una situazione difficile, le conseguenze negative che possono emergere non mi fermano.
20. Se mi accorgo che una persona è in difficoltà, non esito ad aiutarla.
21. **(R)** Spesso sento dolori localizzabili al petto, allo stomaco o ad altri distretti corporei.
22. So anteporre sempre il dovere al piacere.
    1. **FOUND questionnaire – English**

Instructions: The following statements refer to ways of acting, thinking, and feeling that are common in everyday life. Please assign the score that best reflects your experience based on the statement presented.

| 1  Strongly disagree | 2  Disagree | 3  Agree | 4  Strongly agree |
| --- | --- | --- | --- |

1. It is essential to behave properly to avoid exhibiting conduct that may be judged as wrong.
2. feel I am more capable than others at orienting myself in space.
3. After a night’s sleep, I feel filled with strength to face the day.
4. Being able to listen to other people’s reasoning adds value to knowledge.
5. I believe that all people deserve love and care.
6. I perceive my body as flexible and adaptable.
7. I enjoy making others feel good.
8. **(R)** I prefer to delegate to others when I have to monitor actions that involve other people.
9. **(R)** If I find myself in an unfamiliar space without points of reference, I have difficulty in orienting myself.
10. In a group, there needs to be a leader to direct everyone’s tasks.
11. I believe that personal interests should be put aside for the good of the group.
12. Being proactive toward those who suffer is a fundamental pillar of my life.
13. Even when my workload is heavy, I manage to not feel distressed during moments of leisure.
14. If I use a tool, it becomes a natural extension of my body.
15. **(R)** I find it hard to immerse myself in activities I need to perform.
16. Dedication to something means being entirely committed to it, allowing yourself nothing else.
17. I find teamwork gratifying.
18. I believe that obedience is fundamental in life.
19. When I find myself in a difficult situation, the potential negative consequences do not stop me.
20. If I realize that someone is in trouble, I do not hesitate to help them.
21. **(R)** I often feel localized pain in my chest, stomach, or other areas of my body.
22. I can prioritize duty over pleasure.
    1. **FOUND questionnaire – Items and Factors**

| ITEM | FACTOR |
| --- | --- |
| Item_1 | Group-Oriented Values |
| Item_2 | Perception and Action |
| Item_3 | Perception and Action |
| Item_4 | Empathic Attitude |
| Item_5 | Empathic Attitude |
| Item_6 | Perception and Action |
| Item_7 | Empathic Attitude |
| Item_8 | Stress Management |
| Item_9 | Perception and Action |
| Item_10 | Group-Oriented Values |
| Item_11 | Group-Oriented Values |
| Item_12 | Empathic Attitude |
| Item_13 | Stress Management |
| Item_14 | Perception and Action |
| Item_15 | Stress Management |
| Item_16 | Group-Oriented Values |
| Item_17 | Empathic Attitude |
| Item_18 | Group-Oriented Values |
| Item_19 | Stress Management |
| Item_20 | Empathic Attitude |
| Item_21 | Stress Management |
| Item_22 | Stress Management |

# Subgroup results

#### High Procedural Occupations

The repeated-measures ANOVA including only the high-procedural occupations (Creative/Service, Professional/Managerial, Tech) showed a **main effect of FOUND’S factor** (F(3,225)=45.90, p<.001, η²ₚ=.38), but **no significant main effect of High Procedural subgroup** (F(2,75)=0.98, p=.38, η²ₚ=.03) and **no interaction** (F(6,225)=0.56, p=.77, η²ₚ=.01).
Estimated marginal means indicated similar patterns across occupations for all FOUND dimensions (PA, EA, SM, GOV). Bonferroni-corrected pairwise comparisons confirmed no significant contrasts (all p>.10).


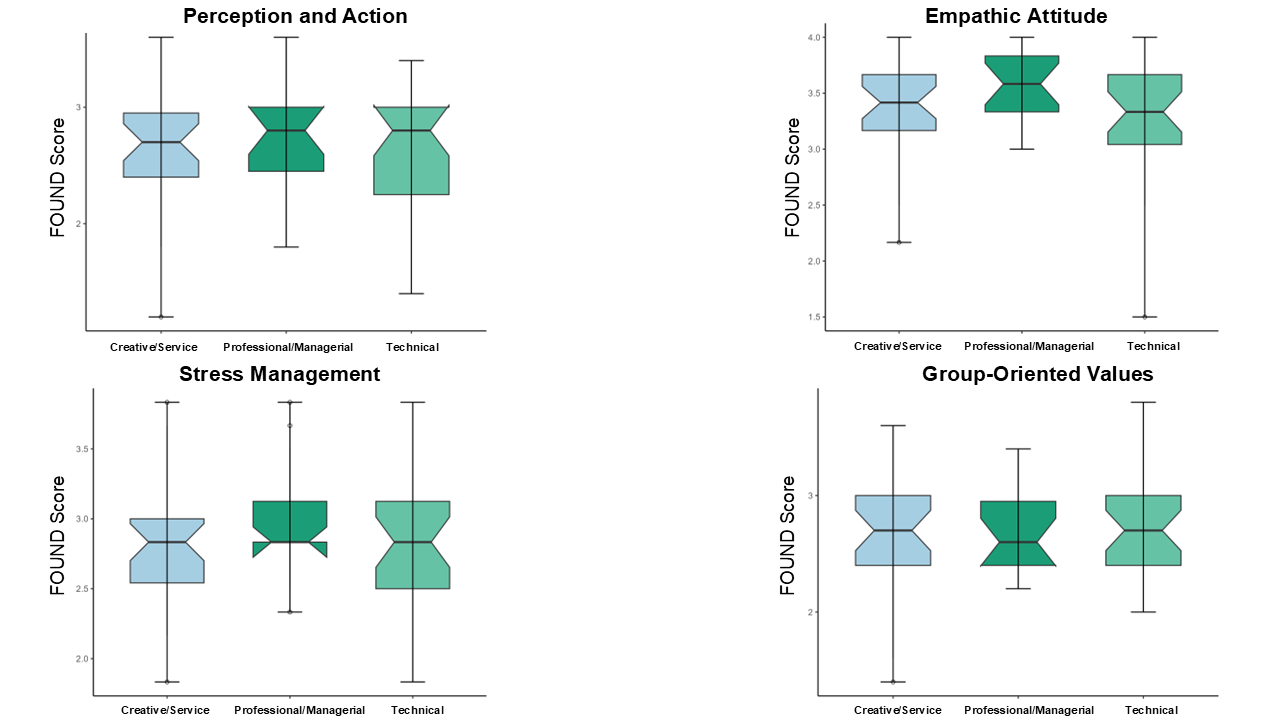


**Supplementary Figure 1.** Comparisons between FOUND scores among High Procedural occupations: Professional Managers, Technical Specialists, Creative Service Professionals.

#### Low Procedural Occupations

The repeated-measures ANOVA including only low-procedural occupations (Administrative, Field-Based), the ANOVA showed a **main effect of FOUND’s factor** (F(3,327)=22.45, p<.001, η²ₚ=.17), but **no main effect of occupational subgroup** (F(1,109)=0.15, p=.70, η²ₚ<.01) and **no interaction** (F(3,327)=0.96, p=.41, η²ₚ<.01).
Pairwise comparisons between the two groups (Administrative vs. Field-Based) were not significant across FOUND dimensions (all p>.29).


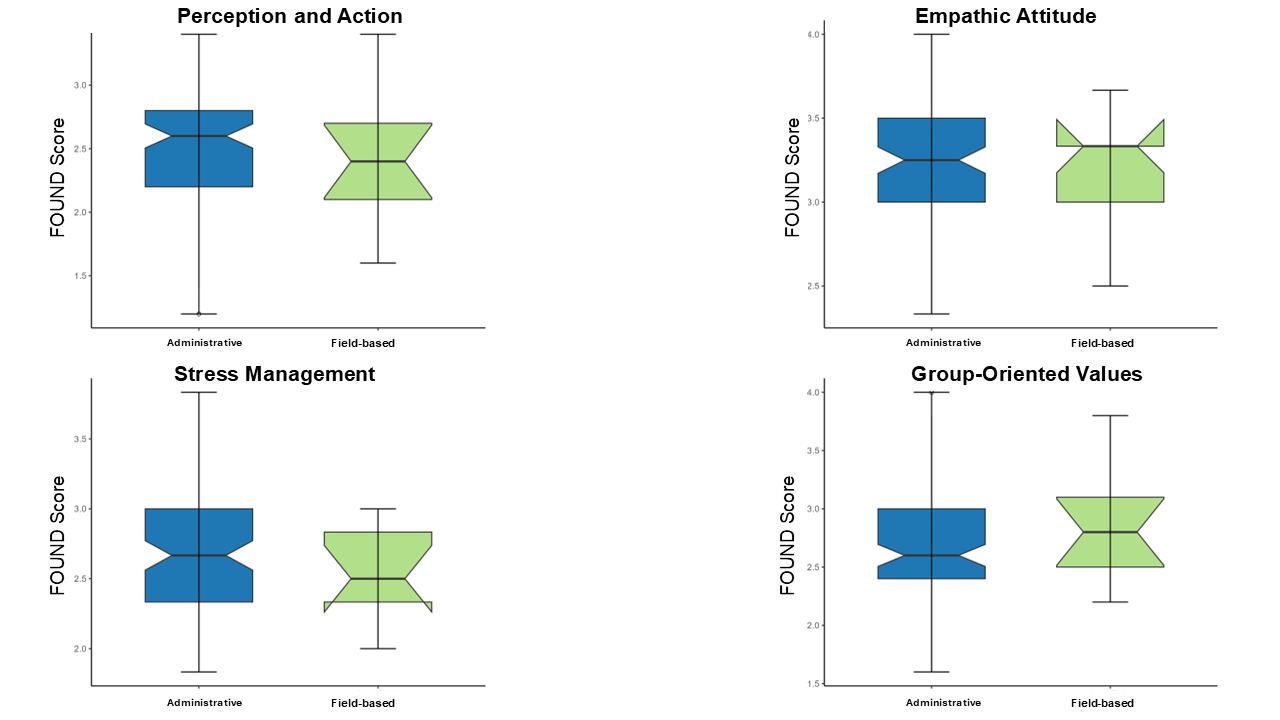


**Supplementary Figure 2.** Comparisons between FOUND scores among Low Procedural occupations: Administrative Staff, Field Workers. .
